# Sensitivity of Allelic Divergence to Genomic Position: Lessons from the *Drosophila tan* Gene

**DOI:** 10.1534/g3.116.032029

**Published:** 2016-07-21

**Authors:** Alisha V. John, Lisa L. Sramkoski, Elizabeth A. Walker, Arielle M. Cooley, Patricia J. Wittkopp

**Affiliations:** * Department of Molecular, Cellular, and Developmental Biology, University of Michigan, Ann Arbor, Michigan 48109; † Department of Ecology and Evolutionary Biology, University of Michigan, Ann Arbor, Michigan 48109

**Keywords:** allele-specific, transgenic analysis, position effect, pigmentation, *tan*

## Abstract

To identify genetic variants underlying changes in phenotypes within and between species, researchers often utilize transgenic animals to compare the function of alleles in different genetic backgrounds. In *Drosophila*, targeted integration mediated by the ΦC31 integrase allows activity of alternative alleles to be compared at the same genomic location. By using the same insertion site for each transgene, position effects are generally assumed to be controlled for because both alleles are surrounded by the same genomic context. Here, we test this assumption by comparing the activity of *tan* alleles from two *Drosophila* species, *D. americana* and *D. novamexicana*, at five different genomic locations in *D. melanogaster*. We found that the relative effects of these alleles varied among insertion sites, with no difference in activity observed between them at two sites. One of these sites simply silenced both transgenes, but the other allowed expression of both alleles that was sufficient to rescue a mutant phenotype yet failed to reveal the functional differences between the two alleles. These results suggest that more than one insertion site should be used when comparing the activity of transgenes because failing to do so could cause functional differences between alleles to go undetected.

Understanding the genetic basis of phenotypic change remains a pressing challenge for evolutionary biology. Addressing this challenge requires the identification of the genes contributing to phenotypic divergence as well as the specific changes within those genes that alter their function (Stern and Orgogozo 2008). Linkage mapping and genome-wide association studies (GWAS) are often used to identify regions of the genome associated with phenotypic divergence (Martin and Orgogozo 2013); however, these approaches must be supplemented with functional tests to demonstrate the phenotypic consequences of individual genes and sequence changes. This functional testing is often accomplished through transgenic analysis that evaluates the effects of a specific gene or region of a gene in different genetic backgrounds.

In *Drosophila*, the activity of divergent alleles is typically compared using transgenes inserted into the genome by transposon-mediated transformation (Wittkopp 2006). Most transposons used for this purpose (*e.g.*, *P-elements*, *piggyBac*, and *Hermes*) insert a transgene semirandomly into the genome (Engels 1996; Garza *et al.*1991; Guimond *et al.* 2003; Handler and Harrell 1999; Smith *et al.* 1993; Spradling and Rubin 1983), which is not ideal because the genomic position of a gene can affect its activity, a phenomenon known as “position effect” (Sturtevant 1925; Wilson *et al.* 1990). These position effects can result from chromatin structure at the insertion site (Huisinga *et al.* 2016; Levis *et al.* 1985; Wilson *et al.* 1990) and/or interactions between the sequence of the transgene and the surrounding DNA that affect expression of the transgene (Venken and Bellen 2007; Wilson *et al.* 1990). The former generally affects the expression level of the transgene, whereas the latter can impact its expression level and/or spatiotemporal regulation. The extent of position effects has been hypothesized to be the product of two variables: (i) the strength of regulatory elements at the genomic location in which the transgene is inserted, and (ii) the susceptibility of the regulatory sequences in the transgene to altered activity (Wilson *et al.* 1990). The addition of insulator sequences flanking a transgene can reduce the effects of surrounding genomic context on its activity (Gdula *et al.* 1996; Kuhn and Geyer 2003; Silicheva *et al.* 2010).

Position effects are especially problematic when comparing activity among transgenes expected to vary in subtle ways. Targeted insertion of transgenes in *Drosophila*, most notably using the bacteriophage ΦC31 integrase system (Groth *et al.* 2004), can help control for position effects by inserting each transgene of interest into the same genomic position of otherwise identical genomes (Venken and Bellen 2005). With large collections of “landing sites” (sequences that mediate integration of the transgene) for ΦC31-mediated transformation available (Bateman *et al.* 2006; Bischof *et al.* 2007; Venken *et al.* 2006), this method has become the standard for comparing the activity of related alleles in *Drosophila*. Typically, such a study compares a set of transgenic lines in which each transgene is integrated independently into a chosen landing site, with a single landing site used in most cases (*e.g.*, Cande *et al.* 2009; Duncan *et al.* 2010; Frankel *et al.* 2010; Haley *et al.* 2010; Joshi *et al.* 2010; Kalay and Wittkopp 2010; Perry *et al.* 2011; Rebeiz *et al.* 2011; Sayal *et al.* 2011). The use of a single landing site for such studies is justified by the assumption that all alleles compared will be affected similarly by the surrounding genomic context (Wimmer 2005). But is this true? Are sets of related transgenes influenced similarly by the surrounding DNA sequence?

Here, we test this assumption by examining the impact of position effects on a comparison of orthologous alleles that contribute to phenotypic divergence between a pair of closely related *Drosophila* species. Specifically, we compare the effects of *tan* alleles from *D. americana* and *D. novamexicana* integrated into the *D. melanogaster* genome at five different genomic locations. *D. americana* and *D. novamexicana* diverged ∼400,000 years ago (Caletka and McAllister 2004; Morales-Hojas *et al.* 2008) and have evolved dramatic differences in adult pigmentation (Throckmorton 1982); *D. americana* has a brown body, whereas *D. novamexicana* has a yellow body (Figure 1). Prior work has shown that these differences in pigmentation are due in part to divergent sites located in the *tan* gene (Wittkopp *et al.* 2009). As described below, we found that position effects influenced whether or not a difference in activity could be detected between these two species-specific alleles of *tan*. Further analysis showed that the ability to detect a difference in activity was related to level of expression from the *tan* transgene at each site. These findings suggest that differences between transgenes should be assessed using multiple landing sites.

**Figure 1:**
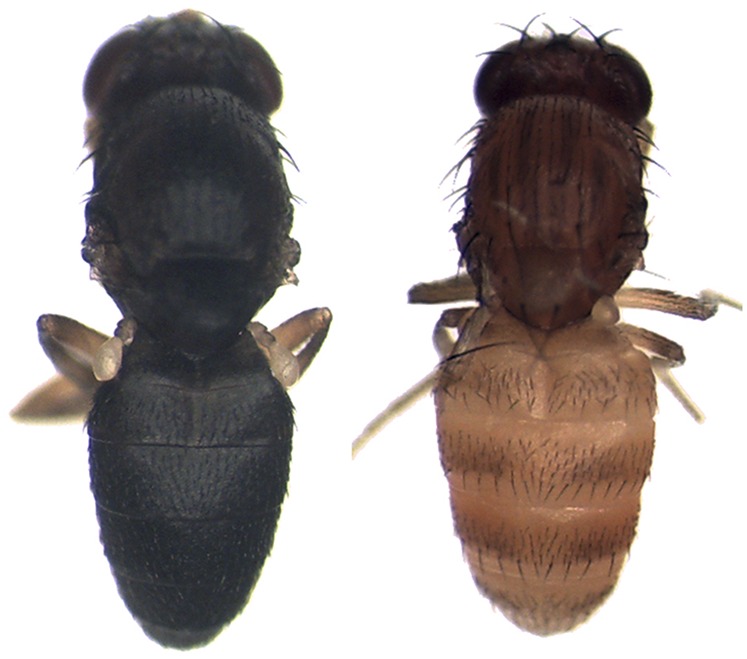
Body color of *D. americana* and *D. novamexicana*. *D. novamexicana* (right) has evolved lighter body pigmentation since it diverged from the common ancestor shared with *D. americana* (left). *D. americana* has retained the darker body pigmentation shared by all other members of the *virilis* group.

## Materials and Methods

### Generation of transgenic flies

Previously constructed transgenes containing *D. americana* or *D. novamexicana tan* (Wittkopp *et al.* 2009) were injected into *D. melanogaster* using the ΦC31 integrase system. The transgenes contained all exonic and intronic sequences of *tan*, as well as 4.1 kb of sequence 5′ of *tan* and 3.6 kb of sequence 3′ of *tan* in a *piggyBac* vector (Horn and Wimmer 2000) containing an attB site used for ΦC31-mediated transformation and Pax6-EGFP, an eye-expressing green fluorescence marker used to detect successful integration (Wittkopp *et al.* 2009). In addition to noncoding and synonymous changes, these *D. americana* and *D. novamexicana tan* transgenes differ by two amino acids; however, these amino acid differences are not fixed between species and are thus unlikely to be responsible for the species-specific differences in pigmentation (Wittkopp *et al.* 2009). Each *D. melanogaster* host genotype carried a transgene on the X-chromosome using the *vasa cis*-regulatory sequences to express the ΦC31 integrase specifically in the germ-line and a single attP site located on the second (51C – BDSC #24482, 58A – BDSC #24484), third (86Fa – BDSC #24486, 86Fb – BDSC #24749), or fourth (102D – BDSC #24488) chromosome (Bischof *et al.* 2007). These lines were selected because they contain an eye-expressing red fluorescent protein (RFP) as a visible marker for the landing site; this is in contrast to the majority of strains containing attP landing sites that are commonly used, which use a copy of the *yellow* gene (which restores dark pigmentation in *yellow* mutant flies) as a visible marker for the landing site. GenetiVision (Houston, Texas) performed all DNA preparations and embryo injections according to their standard protocols (http://www.genetivision.com/). Transformant flies (expressing green fluorescent protein in their eyes) were used to establish lines homozygous for each transgene in a *D. melanogaster* background carrying loss-of-function mutations in the X-linked genes *tan*, *yellow*, and *white*. The mutant *D. melanogaster tan* allele allowed us to test for rescue of the *tan* mutant pigmentation phenotype by the heterologous *tan* alleles contained in the transgenes; the *yellow* mutant allele reduced the amount of black pigment present in these flies, providing a more sensitive assay for changes in abdominal pigmentation caused by the transgenes; and the *white* allele allowed for easier visualization of the eye-expressing fluorescent transformation marker.

### Drosophila husbandry, collection, and abdominal cuticle dissection

For each line to be analyzed, virgin females were mated with males on standard yeast-glucose media at 20°. Upon formation of pupae, parents were removed and the offspring were allowed to continue development. Male offspring were collected 0–1 d posteclosion and aged to 7–8 d. Flies were stored in 10% glycerol in ethanol prior to dissection.

To harvest abdominal cuticles, 7–8-day-old males were removed individually from the 10% glycerol in ethanol solution and placed on a glass slide. Using a razor blade, the abdomen was separated from the rest of the body then cut along the lateral edge parallel with the anterior-posterior axis. The dorsal half of the abdomen was soaked overnight in a solution of phosphate buffered saline (PBS; 1.4 M NaCl, 27 mM KCl, 190 mM Na_2_HPO_4_, and 18 mM KH_2_PO_4_, adjusted to pH 7.4 with 1 M HCl). After soaking overnight, a single dorsal half of abdominal cuticle was removed from the PBS and placed on a glass slide, dorsal (cuticle) side down. Using forceps, the abdominal cuticle was cleared of any remaining debris. The cleaned cuticle was then mounted dorsal side up in polyvinyl alcohol (PVA) mounting media (BioQuip) on a clean glass slide, covered with a coverslip, and the coverslip was sealed with clear nail polish. This process was repeated for all genotypes analyzed, with 17–35 (mean = 27) flies analyzed for each genotype. To minimize effects of any day-to-day differences in dissections, all genotypes were dissected during each dissection session.

### Image collection and processing

Dissected abdominal cuticles were imaged in a single session using a Leica MZ6 microscope and Scion (CFW-1308C) camera operated via TWAIN driver in Adobe Photoshop. Magnification was set to 3.2 with ring light illumination at ∼75%. At the beginning of the imaging session, auto white balance (AWB) was used, resulting in a configuration of Gamma 0.605, Red Gain -1.4 db, Green Gain 5.4 db, and Blue Gain 8.9 db with Red Boost and Blue Boost active. These settings were not changed throughout the imaging session. Imaging was conducted at night to minimize changes in ambient lighting. Images were taken slide-by-slide (2 cuticles/slides, cuticles imaged individually) with samples arranged such that no more than two cuticles from the same line were imaged consecutively. A “reference” image of the same dissected cuticle was taken approximately every 10 slides to allow us to evaluate the consistency of the image collection, processing, and analysis pipeline during the multi-hour imaging session.

All images were compiled into a single document in Adobe Photoshop and the “Levels” function was used to adjust the color of all images simultaneously so that the images more closely matched the cuticle appearance visible by eye. This ensured that an identical color adjustment was applied to all photos.

### Quantifying pigmentation

**Figure 2:**
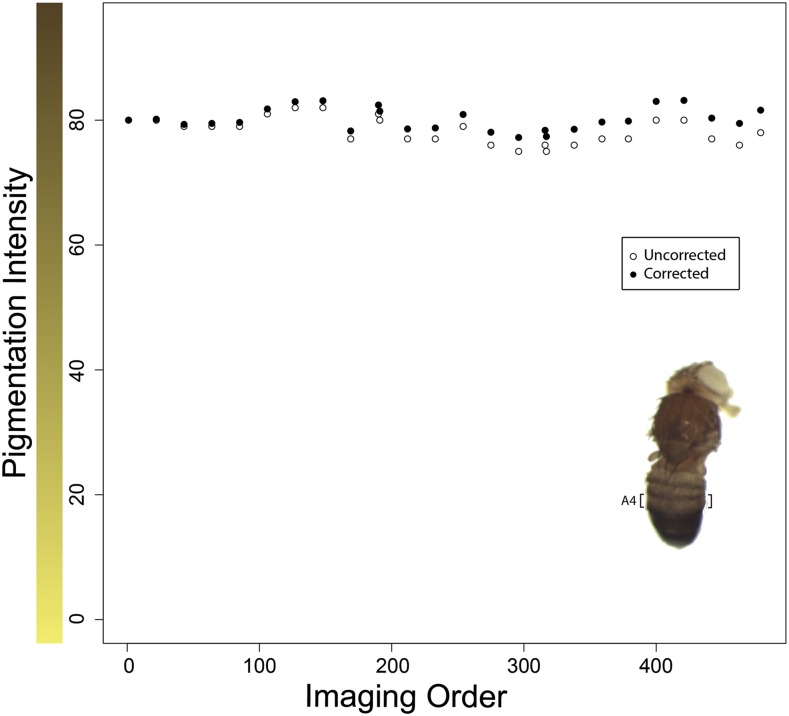
Measurements of pigmentation intensity in a control sample varied slightly during image collection. Raw median pigmentation intensity in tergite A4 (insert) is plotted against imaging order for the reference cuticle (open circles). All images were taken during in a single sitting without adjustment of lighting, focus, or other imaging parameters; the small (β = −0.0075), yet significant (p-value = 0.008), downward trend in pigmentation intensity as the imaging progressed, presumably as a result of changes in ambient lighting or other uncontrolled imaging variables. An imaging order correction was therefore applied to all measurements, as described in the *Materials and Methods* section. Corrected median pigmentation intensity values for the same images are also plotted against imaging order (closed circles) to show the effects of this correction.

Using ImageJ (Rasband 1997-2016), the area of dorsal abdominal cuticle known as abdominal tergite 4, or “tergite A4,” (insert, Figure 2) was manually selected using the polygon tool, excluding any regions containing cracks, holes, or overlapping regions. Measurements of pixel intensity (area, mean, standard deviation, mode, min, max, and median) were taken for each selection. These results were compiled into a single Microsoft Excel spreadsheet where other identifying information was then added, including imaging order (ranging from 1 to 479), allele (no transgene control, *D. americana*, and *D. novamexicana*), and landing site (control, 51C, 58A, 86Fa, 86Fb, and 102D). Since ImageJ quantifies pigmentation (pixel intensity of a grayscale image) on a 0–255 scale (dark–light), we subtracted the reported pixel intensity from 255 so that darker cuticles had a higher pigmentation score. This file was then saved as a .csv file for statistical analysis in R.

### Data analysis

Median pigmentation intensity of tergite A4 for each sample reported by ImageJ was analyzed using R v3.2.5 (R Development Core Team 2016). Median pigmentation was chosen for analysis instead of mean pigmentation intensity to minimize the impact of outlier (excessively white or black) pixels.

To test for systematic changes in imaging conditions that might have occurred during the imaging session, a linear regression was performed comparing median pigmentation values from the reference cuticle and the image order number. A small but significant regression coefficient (β = −0.0075, p-value = 0.008) was observed, so a correction for imaging order was applied to each median by subtracting (image order number * −0.0075) from the original median value. The differences in reference cuticle values before and after applying this correction are shown in Figure 2. Note that all analyses described below were also performed on data without this correction and produced the same pattern of statistically significant results (data not shown).

Median pigmentation intensity of tergite A4 for each sample reported by ImageJ was then fitted to the following model to test effects of landing site, allele, and the interaction between the two:

$$Y_{ijk}=site_{i}+allele_{j}+site\times allele_{ij}\;+\;e_{ijk}$$

Pairwise *t*-tests using unpooled standard deviations were then performed on the corrected pigmentation medians to identify which comparisons among *tan* alleles and/or landing sites were statistically significant. Statistical significance was assessed using p-values adjusted for multiple testing by the Benjamini and Hochberg method (Benjamini and Hochberg 1995) as implemented in the pairwise.t.test function in R.

### Measuring relative expression of tan transgenes at each genomic location

To test for differences in the expression level of transgenes inserted at each genomic location in *D. melanogaster*, relative expression levels of the *D. americana tan* transgene were measured using pyrosequencing (Wittkopp 2011). Specifically, we measured the mRNA abundance of the *D. americana tan* allele inserted at each genomic location relative to the mRNA abundance of the *D. novamexicana tan* allele inserted at the 86Fa landing site. This *D. novamexicana* genotype was chosen as the internal reference point for measurements of *D. americana tan* expression because it caused an intermediate pigmentation phenotype, suggesting it might also have an intermediate level of expression. The P14–P15 pupal stage was analyzed because *D. americana* and *D. novamexicana tan* have previously been shown to be most highly expressed during this time (Cooley *et al.* 2012). Pupal heads and wings were removed to avoid measuring *tan* expression in those tissues, focusing our measurements on expression in the thorax and abdomen where pigmentation phenotypes are most apparent.

For each landing site, both genomic DNA and total RNA were extracted from three replicate samples, each containing six dissected pupae expressing *D. americana tan* and six dissected pupae expressing *D. novamexicana tan*. cDNA was reverse transcribed from extracted RNA using a polyT primer for each sample. Both genomic DNA and cDNA were analyzed by pyrosequencing as described in Wittkopp (2011). PCR primers used to amplify the sequence analyzed (which was located in exon 7) were 5′-GATGCTGAAGTCCAGCGTGTC-3′ and 5′-biotin-CAGCCGCCAGTGACATCA-3′, and the primer used for pyrosequencing had the sequence 5′-CGAGCACGATGTCCG-3′. All measurements were then normalized to the relative expression of the *D. americana tan* transgene inserted at landing site 86Fa to compare expression among the D. *americana tan* transgenes at different landing sites.

### Supplemental files

Supplemental Material, File S1 contains pigmentation measures from all individual cuticles. File S2 contains the code used to analyze the pigmentation measures. File S3 contains the raw and analyzed pyrosequencing data.

### Data availability

The authors state that all data necessary for confirming the conclusions presented in the article are represented fully within the article.

## Results and Discussion

To test the assumption that position effects are negligible when comparing divergent alleles of the same gene at a single genomic location, we transformed *D. americana* and *D. novamexicana tan* alleles into five different genomic locations in *D. melanogaster* (51C, 58A, 86Fa, 86Fb, and 102D). Each of these transgenic lines was then crossed with *D. melanogaster yellow*, *white*, and *tan* mutants (see *Materials and Methods* for full genotype) to move the transgenes into genetic backgrounds lacking a functional copy of the *D. melanogaster tan* gene. Prior work has shown that the difference in body color seen between *D. americana* and *D. novamexicana* (Figure 1) is due in part to changes in *tan* and that these *D. americana* and *D. novamexicana tan* transgenes significantly increase abdominal pigmentation in a *D. melanogaster tan* mutant (Wittkopp *et al.* 2009). The transgenic *tan* allele from the more darkly pigmented *D. americana* was reported to increase pigmentation significantly more than the transgenic *tan* allele from the more lightly pigmented *D. novamexicana*, indicating that there is functional divergence between these species-specific alleles that affects pigmentation (Wittkopp *et al.* 2009).

To determine whether the insertion site of the *D. americana* and *D. novamexicana tan* transgenes affected their relative activity, we used an analysis of variance (ANOVA) to test for significant effects on pigmentation of allelic identity of the *tan* transgene (*D. americana* or *D. novamexicana*), genomic location of the landing site, and the interaction between the two. All three factors were found to be statistically significant predictors of pigmentation intensity (Table 1). In other words, pigmentation differences were detected between alleles and among landing sites, and the difference between alleles differed among landing sites. The significance of this interaction term is particularly interesting because it suggests that the effects of genomic context might differ between alleles, implying that the landing site used to compare the function of *D. americana* and *D. novamexicana* alleles might alter the conclusions drawn about differences (or lack thereof) between these two alleles.

**Table 1:** Analysis of variance (ANOVA) in pigmentation indicates that *tan* allelic identity, genomic location, and the interaction between allele and genomic location affect pigmentation intensity

Source of Variation	Degrees of Freedom	Sum of Squares	Mean Square	F value (F_0_)	p-Value
1) *tan* transgene identity	2	3.08 × 10^4^	1.54 × 10^4^	1.54 × 10^2^	2.76 × 10^−46^
2) Landing site	4	5.74 × 10^4^	1.44 × 10^4^	1.43 × 10^2^	1.89 × 10^−67^
3) Interaction between 1 and 2	4	2.54 × 10^3^	6.34 × 10^2^	6.33	6.73 × 10^−5^
4) Residuals	290	2.90 × 10^4^	90.5	N/A	N/A

N/A, not applicable.

One way that the genomic context can affect a transgene is to simply silence it. To determine whether such silencing was contributing to the difference in allelic differences observed among insertion sites, we used *t*-tests to determine whether each transgene caused a statistically significant darkening of pigmentation in each transgenic line relative to the *D. melanogaster tan* mutant phenotype. Such a darkening would indicate that the transgene carried was being expressed at a level sufficient to restore at least some dark pigmentation in *D. melanogaster*. We found that the transgenic *tan* alleles from both *D. americana* and *D. novamexicana* failed to significantly alter pigmentation of the *D. melanogaster tan* mutant when inserted into the fourth chromosome at cytological position 102D (Table 2). This evidence of transgene silencing is consistent with prior studies showing that the fourth chromosome of *D. melanogaster* is highly heterochromatic (Riddle and Elgin 2006; Riddle *et al.* 2009) and can suppress the expression of transgenes (Salzler *et al.* 2013; Sun *et al.* 2000). Landing site 102D does not always silence transgenes, however; other transgenes inserted into the 102D landing site have been shown to be expressed during larval stages (Bischof *et al.* 2007; Barolo and Evans, personal communication). At each of the other four landing sites tested (all located on chromosome 2 or chromosome 3), both the *D. americana* and *D. novamexicana tan* transgenes caused a significant darkening of pigmentation relative to the *tan* mutant phenotype (Table 2), indicating that the transgenes were expressed and producing functional Tan protein. To determine whether the silencing of transgenes at landing site 102D was sufficient to explain the significant interaction observed between transgene identity and landing site in the initial ANOVA, we excluded flies with transgenes inserted into this site and repeated this ANOVA. We found that the two main effect terms (transgene identity and landing site) and the interaction term remained statistically significant (Table 3), indicating that the relative activity of the *D. americana* and *D. novamexicana tan* transgenes differed even among sites that allowed expression of both transgenes.

**Table 2:** Pairwise t-tests show which transgenes inserted at which insertion sites alter pigmentation relative to *D. melanogaster tan* mutants, as well as which landing sites show evidence of functional differences between the *D. americana* and *D. novamexicana tan* alleles

			51C	51C	58A	58A	86Fa	86Fa	86Fb	86Fb	102D
		*tan* mutant	*D. amer*	*D. nova*	*D. amer*	*D. nova*	*D. amer*	*D. nova*	*D. amer*	*D. nova*	*D. amer*
51C	*D. amer*	4 × 10^−9^	—	—	—	—	—	—	—	—	—
51C	*D. nova*	8 × 10^−8^	0.411	—	—	—	—	—	—	—	—
58A	*D. amer*	5 × 10^−18^	3 × 10^−10^	2 × 10^−11^	—	—	—	—	—	—	—
58A	*D. nova*	4 × 10^−12^	1 × 10^−3^	1 × 10^−4^	5 × 10^−5^	—	—	—	—	—	—
86Fa	*D. amer*	4 × 10^−24^	3 × 10^−14^	1 × 10^−15^	0.317	2 × 10^−7^	—	—	—	—	—
86Fa	*D. nova*	2 × 10^−21^	4 × 10^−7^	1 × 10^−8^	1 × 10^−3^	0.093	2 × 10^−6^	—	—	—	—
86Fb	*D. amer*	3 × 10^−23^	1 × 10^−15^	9 × 10^−17^	0.012	2 × 10^−9^	0.064	1 × 10^−8^	—	—	—
86Fb	*D. nova*	9 × 10^−17^	1 × 10^−7^	7 × 10^−9^	0.064	0.011	2 × 10^−3^	0.195	2 × 10^−5^	—	—
102D	*D. amer*	0.125	1 × 10^−4^	1 × 10^−3^	9 × 10^−14^	3 × 10^−8^	4 × 10^−15^	8 × 10^−11^	9 × 10^−17^	1 × 10^−11^	—
102D	*D. nova*	0.088	3 × 10^−7^	7 × 10^−6^	7 × 10^−17^	8 × 10^−11^	2 × 10^−23^	3 × 10^−20^	3 × 10^−22^	2 × 10^−15^	0.706

p-values adjusted by the Benjamini and Hochberg method from all possible pairwise *t*-tests using unpooled standard deviation are shown for each pair of genotypes compared. The *tan* mutant column shows results from comparisons between each transgenic genotype and the *tan* mutant (no transgene) control. Note that neither transgene darkened pigmentation relative to the *tan* mutant when inserted at landing site 102D. Highlighted boxes indicate comparisons between the *D. americana* and *D. novamexicana tan* alleles inserted at the same landing site. Significant differences (*p* < 0.05) in median pigmentation were observed for transgenes inserted at 58A, 86Fa, and 86Fb, but not 51C or 102D. *D. amer*, *D. Americana*; *D. nova*, *D. novamexicana*.

**Table 3:** Interaction between allelic identity and genomic location remains significant after excluding silenced transgenes

Source of Variation	Degrees of Freedom	Sum of Squares	Mean Square	F Value (F_0_)	p-Value
1) *tan* transgene identity	2	3.53 × 10^4^	1.77 × 10^4^	1.66 × 10^2^	6.39 × 10^−46^
2) Landing site	3	2.41 × 10^4^	8.05 × 10^3^	75.5	2.00 × 10^−34^
3) Interaction between 1 and 2	3	1.54 × 10^3^	5.13 × 10^2^	4.82	2.83 × 10^−3^
4) Residuals	240	2.56 × 10^4^	1.07 × 10^2^	N/A	N/A

Results from analysis of variance (ANOVA) after excluding flies with transgenes inserted at landing site 102D are shown. N/A, not applicable.

**Figure 3:**
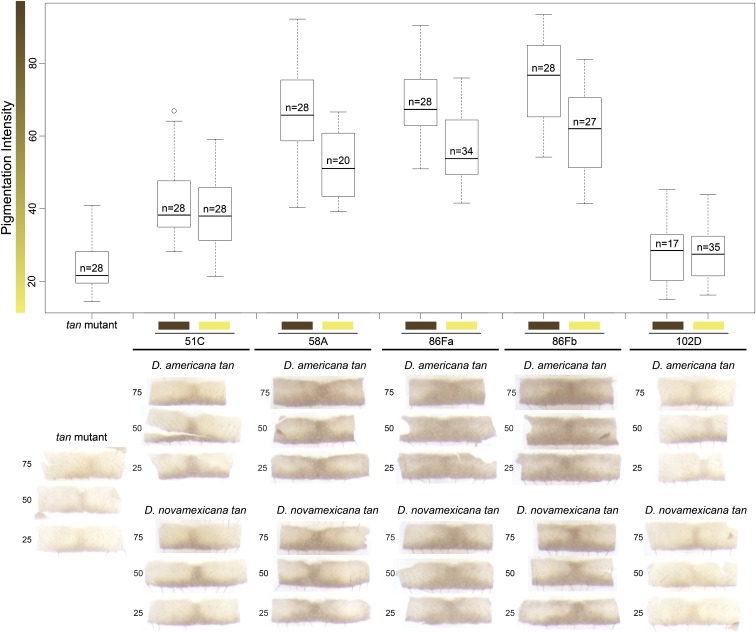
Genomic location can impact the relative difference in pigmentation caused by *D. americana* and *D. novamexicana tan* alleles. Box plots of pigmentation intensity summarize, for each genotype, the range of pigmentation phenotypes observed. The median (center line), first quartile (bottom of box), third quartile (top of box), and ± 1.5 × the interquartile range (whiskers) are shown for each genotype examined. Yellow boxes along the x-axis represent the *D. novamexicana* allele and brown boxes represent the *D. americana* allele. Significant increases in pigmentation from the control were detected for all genomic locations except 102D (Table 2). Three of the other four landing sites (58A, 86Fa, and 86Fb) showed significant differences in pigmentation driven by the *D. americana* and *D. novamexicana tan* alleles, whereas the fourth landing site (51C) did not show a detectable difference in pigmentation between flies carrying the two species’ alleles (Table 2). Representative images from the 25th percentile (first quartile), median, and 75th percentile (third quartile) are shown below the box plot for each genotype. The most striking differences between alleles are seen in the anterior regions outside the dorsal midline stripe.

To further investigate this difference in relative transgene activity among insertion sites, we used a series of *t*-tests to compare the pigmentation phenotype caused by the *D. americana* and *D. novamexicana tan* alleles inserted at the same landing site. We found that the *D. americana tan* allele increased dark pigmentation of the *D. melanogaster tan* mutant significantly more than the *D. novamexicana tan* allele when inserted at three (58A, 86Fa, and 86Fb) of the four landing sites expressing the transgenes (Figure 3 and Table 2). The difference in activity between these two alleles was masked, however, when then transgenes were inserted into the landing site at 51C (*P* = 0.411, Figure 3 and Table 2). Excluding flies with transgenes at this landing site (51C) as well as flies with transgenes at the landing site that silenced the transgenes (102D) from the ANOVA described above resulted in a nonsignificant interaction between transgene allele and insertion site (Table 4), indicating that the relative effects of the *D. americana* and *D. novamexicana tan* transgenes on pigmentation were comparable at the 58A, 86Fa, and 86Fb landing sites.

**Table 4:** Differences in pigmentation between flies carrying the *D. americana* and *D. novamexicana tan* transgenes are similar among the three landing sites that showed a significant difference between transgenes

Source of Variation	Degrees of Freedom	Sum of Squares	Mean Square	F Value (F_0_)	p-Value
1) *tan* transgene identity	2	4.58 × 10^4^	2.29 × 10^4^	2.13 × 10^2^	6.11 × 10^−49^
2) Landing site	2	1.85 × 10^3^	9.27 × 10^2^	8.66	2.52 × 10^−4^
3) Interaction between 1 and 2	2	27.8	13.9	0.13	0.88
4) Residuals	186	1.88 × 10^4^	1.07 × 10^2^	N/A	N/A

Results from analysis of variance (ANOVA) after excluding flies with transgenes inserted at landing site 102D and 51C are shown. N/A, not applicable.

**Figure 4:**
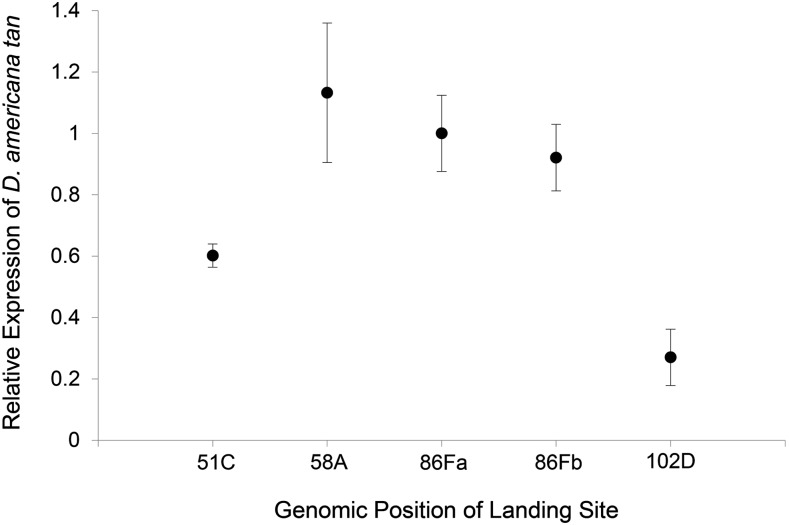
Genomic location impacts relative expression of the *D. americana tan* transgene in *D. melanogaster*. Expression of the *D. americana tan* transgene inserted at each of the five landing sites tested is shown relative to its expression when inserted in the 86Fa landing site. Circles indicate mean expression among replicate samples and the error bars show the 95% C.I. of the estimates. Note that the relative expression level of *D. americana tan* among landing sites correlates with the ability to detect differences in abdominal pigmentation (Figure 3). The *D. americana tan* transgene inserted at 58A, 86Fa, and 86Fb all showed similar expression as well as similar pigmentation phenotypes. The D. *americana tan* transgene inserted at 51C had a level of expression between these lines and the line with the transgene inserted at 102D, as well as pigmentation that was intermediate between these lines and 102D. The *D. americana tan* transgene inserted at 102D had the lowest transgene expression and failed to increase dark pigmentation relative to the *tan* mutant phenotype.

Prior work has shown that position effects often alter expression levels of transgenes (*e.g.*, Markstein *et al.* 2008; Namciu *et al.* 1998; Wilson *et al.* 1990), thus we hypothesized that the different pigmentation phenotypes resulting from different insertion sites of the transgenes might be caused by differences in transgene expression among landing sites. To test this hypothesis, we used pyrosequencing to measure the relative expression of the *D. americana tan* transgene among landing sites (Figure 4). Genomic locations (58A, 86Fa, and 86Fb) that showed statistically significant differences in pigmentation caused by the *D. americana* and *D. novamexicana tan* alleles had the highest levels of *D. americana tan* expression. The genomic location (51C) in which the *D. americana* and *D. novamexicana tan* alleles showed a significant increase in pigmentation relative to the *D. melanogaster tan* mutant, but no differences in pigmentation between flies carrying the *D. americana* and *D. novamexicana tan* alleles, had a lower level of *D. americana tan* expression. The genomic location (102D), in which neither the *D. americana* nor the *D. novamexicana tan* transgene increased pigmentation significantly relative to the *D. melanogaster tan* mutant, showed the lowest expression of *D. americana tan* among all five lines. These results confirm that different landing sites resulted in different levels of transgene expression and suggest that a threshold in transgene expression level must be reached before the different activities of the *D. americana* and *D. novamexicana tan* transgenes can be detected. We expect that this will be generally true when comparing activities of divergent alleles inserted into the same genomic location, but that the value of this threshold will likely differ depending on the strength of regulatory sequences in the transgenes, genomic context, and/or the relative difference in activity between alleles.

In summary, by comparing activities of divergent alleles of the same gene at five different genetic locations, we were able to test the assumption that position effects can be ignored as long as the two alleles compared are inserted into the same genomic location and the transgenes are expressed. We found this not to be true; *D americana* and *D. novamexicana tan* transgenes inserted at landing site (51C) increased dark pigmentation relative to a *D. melanogaster tan* mutant, yet showed no significant difference in their relative activity. If we had only compared the effects of these *tan* alleles at the 51C landing site, we would have concluded that they had conserved functions. The lower level of transgene expression at this site relative to transgenes inserted at the three landing sites that allowed a functional difference between the *D. americana* and *D. novamexicana tan* alleles to be detected suggests that landing sites allowing the highest levels of transgene expression might provide the most power for detecting differences between alleles. We recommend that at least three genomic locations should be tested to search for allelic differences in activity. Although this increased production of transgenic lines would increase cost and workload, they would help prevent inaccurate conclusions from being drawn from transgenes affected by position effects.

## Supplementary Material

Supplemental Material
