# Supplementary material for: Sensitivity of Allelic Divergence to Genomic Position: Lessons from the Drosophila tan Gene
Source: G3 (Bethesda). 2016 Jul 21;6(9):2955–62. doi: 10.1534/g3.116.032029 (PMC5015952; doi:10.1534/g3.116.032029)
Supplement: Supplemental Material [file supp_6_9_2955__index.html]

Sensitivity of Allelic Divergence to Genomic Position: Lessons from the Drosophila tan Gene — Sensitivity of Allelic Divergence to Genomic Position: Lessons from the Drosophila tan Gene — Supplemental Material 

# Sensitivity of Allelic Divergence to Genomic Position: Lessons from the *Drosophila tan* Gene

## Supplemental Material for John *et al.*, 2016

**Files in this Data Supplement:**

- File S1 - This file contains pigmentation measures from all individual cuticles. (.csv, 25 KB)
- File S2 - This file contains the code used to analyze the pigmentation measures. (.zip, 2 KB)
- File S3 - This file contains the raw and analyzed pyrosequencing data. (.zip, 26 KB)
